# President's address: 20 years of perspiration and 20 years of admiration

**DOI:** 10.3352/jeehp.2012.9.1

**Published:** 2012-01-03

**Authors:** Kun Sang Kim

**Affiliations:** National Health Personnel Licensing Examination Board, Seoul, Korea.

Of all the goals listed in last year's President's address, most of them were realized. The National Health Personnel Licensing Examination Board (NHPLEB) of the Republic of Korea earned the ISO 9001 certificate in August 2011. The Journal of Educational Evaluation for Health Professions (JEEHP) was accepted as an official journal of the Association for Medical Education in the Western Pacific Region. This means that a new international window was opened for communicating ideas on educational evaluation within the Western Pacific Region. Another remarkable project was two trial runs of an examination of ubiquitous-based testing (UBT) for medical technologist students in November and December 2011. Smart pads were kindly provided by Korea Telecom. Some bandwidth problems and procedural mistakes in the first examination were corrected in the second one. The response from examinees and the faculty was favorable to UBT suggesting the possibility of introducing UBT to the variety of national health personnel licensing examinations. At last November's meeting of the Board of the Korean Medical Licensing Examination, the NHPLEB was also asked to prepare for the introduction of computer-based testing (CBT) within 7 years. 2012 is the 20th anniversary of NHPLEB. To continue the success of the last 20 years of hard work and perspiration, a conference will be held this May. Here, the new short-term and long-term goals of NHPLEB and new educational evaluation projects will be discussed in depth, preparing plans for the next 20 years worthy of our pride and admiration. Among those planned projects, UBT and CBT will be implemented to two health personnel fields, medical technologists and dentists as trial run of examination.

I have been pleased to observe the increase in the papers published through this journal. In 2012, I hope an even greater number of invaluable articles will appear in the journal to promote higher quality educational evaluation for health personnel.

